# X-linked Thrombocytopenia with Normal Wiskott-Aldrich Syndrome Protein Expression in Lymphocytes and a Novel Wiskott-Aldrich Syndrome Protein Gene Variant: A Case Report and Brief Review of the Literature

**DOI:** 10.1016/j.jpedcp.2024.200128

**Published:** 2024-10-10

**Authors:** Serena Hamanaka, Toru Uchiyama, Tadashi Kaname, Motohiro Matsui, Hiroshi Yoshihashi, Atsushi Makimoto, Yuki Yuza, Akira Ishiguro

**Affiliations:** 1 Department of General Pediatrics, Tokyo Metropolitan Children's Medical Center, Tokyo, Japan; 2 Department of Human Genetics, National Center for Child Health and Development, Tokyo, Japan; 3 Department of Genome Medicine, National Center for Child Health and Development, Tokyo, Japan; 4 Department of Hematology/Oncology, Tokyo Metropolitan Children's Medical Center, Tokyo, Japan; 5 Department of Clinical Genetics, Tokyo Metropolitan Children's Medical Center, Tokyo, Japan; 6 Center for Postgraduate Education and Training, National Center for Child Health and Development, Tokyo, Japan

**Keywords:** X-linked thrombocytopenia, Wiskott-Aldrich syndrome protein, novel variant, lymphocyte

## Abstract

We present a case of X-linked thrombocytopenia (XLT) with a novel WAS gene variant expressing a normal amount of Wiskott-Aldrich syndrome protein (WASp) in lymphocytes. XLT usually decreases WASp expression not only in platelets, but also in lymphocytes. However, there were cases, such as the present one, in which WASp was expressed normally in lymphocytes and absent only in platelets. Our finding suggests that it is of greater diagnostic sensitivity to perform an expression analysis of WASp in both platelets and lymphocytes when XLT is suspected.

Wiskott-Aldrich syndrome (WAS) is an inherited, X-chain disease characterized by immunodeficiency, microthrombocytopenia, and eczema.^1^ WAS arises from variants in the gene encoding the WAS protein (WASp). More than 300 *WAS* gene variants have been reported to date.^2^ The genotypes of the *WAS* gene variant influence the degree of WASp expression and may influence the clinical phenotypes.^3^ Clinical phenotypes are classified into three different types: classic WAS, X-linked thrombocytopenia (XLT), and X-linked neutropenia.^4^

In classical WAS, WASp is often absent in both lymphocytes and platelets, whereas in XLT, its expression is usually decreased in lymphocytes^1^ and absent in platelets.^5^ We present a case of a child with the XLT with normal WASp expression in lymphocytes. Moreover, the novel *WAS* gene variant seemed to be responsible for the disease process in the present case.

## Case Report

A 3-year-old patient presented with petechiae on the skin and severe thrombocytopenia on a blood test. He had no history of eczema, recurrent infection, autoimmunity, or malignancy. He had no family history of a bleeding tendency or recurrent infections. Physical examination revealed no abnormal findings other than intraoral petechiae and cutaneous petechiae, and ecchymoses. His platelet count was 22 × 10^9^/L, neutrophil count was 1.9 × 10^9^/L, and the mean platelet volume was 8.2 fL. A peripheral blood smear demonstrated small to normally sized platelets. Although an intravenous injection of immunoglobulin (IVIG 1 g/kg) was administered for a tentative diagnosis of immune thrombocytopenia following the American Society of Hematology 2019 guidelines,^6^ the platelet count failed to increase. Moreover, the neutrophil count gradually decreased from the normal value to 0.5 × 10^9^/L on day 4 after IVIG administration. A bone marrow examination revealed normocellularity with decreased megakaryocytes. Immunological tests demonstrated IgG 999 mg/dL (reference range, 948-1129 mg/dL), IgA 80 mg/dL (reference range, 62-86 mg/dL), and IgM 129 mg/dL (reference range, 116-147 mg/dL). T and B lymphocytes and natural killer cells were 2.28 × 10^9^/L (reference range, 1.40-3.70 × 10^9^/L), 0.05 × 10^9^/L (reference range, 0.39-1.40 × 10^9^/L), and 0.21 × 10^9^/L (reference range, 0.13-0.72 × 10^9^/L), respectively. CD3^＋^CD4^＋^, CD3^＋^CD8^＋^, and CD4/CD8 were 1.11 × 10^9^/L (reference range, 0.70-2.20 × 10^9^/L), 0.63 × 10^9^/L (reference range, 0.49-1.30 × 10^9^/L), and 1.76 (0.40-2.30), respectively.

On day 10 after IVIG administration, the patient's platelet count and neutrophils increased to 40 × 10^9^/L and 0.6 × 10^9^/L, respectively. Although this trend toward recovery continued for some time, both platelets and neutrophils started decreasing after 2 months. Although the neutrophils gradually normalized, the platelets continued to decrease to approximately 20 × 10^9^/L.

As a part of an evaluation for congenital thrombocytopenia, a target sequencing panel was performed for congenital thrombocytopenia after obtaining the parents’ informed consent.^7^ Genetic testing revealed a missense variant of the *WAS*, c.1075 C > A, p.(Pro359Thr) (Figure, A), which had not been reported previously. His mother was a heterozygous carrier of the variant.

**Figure:**
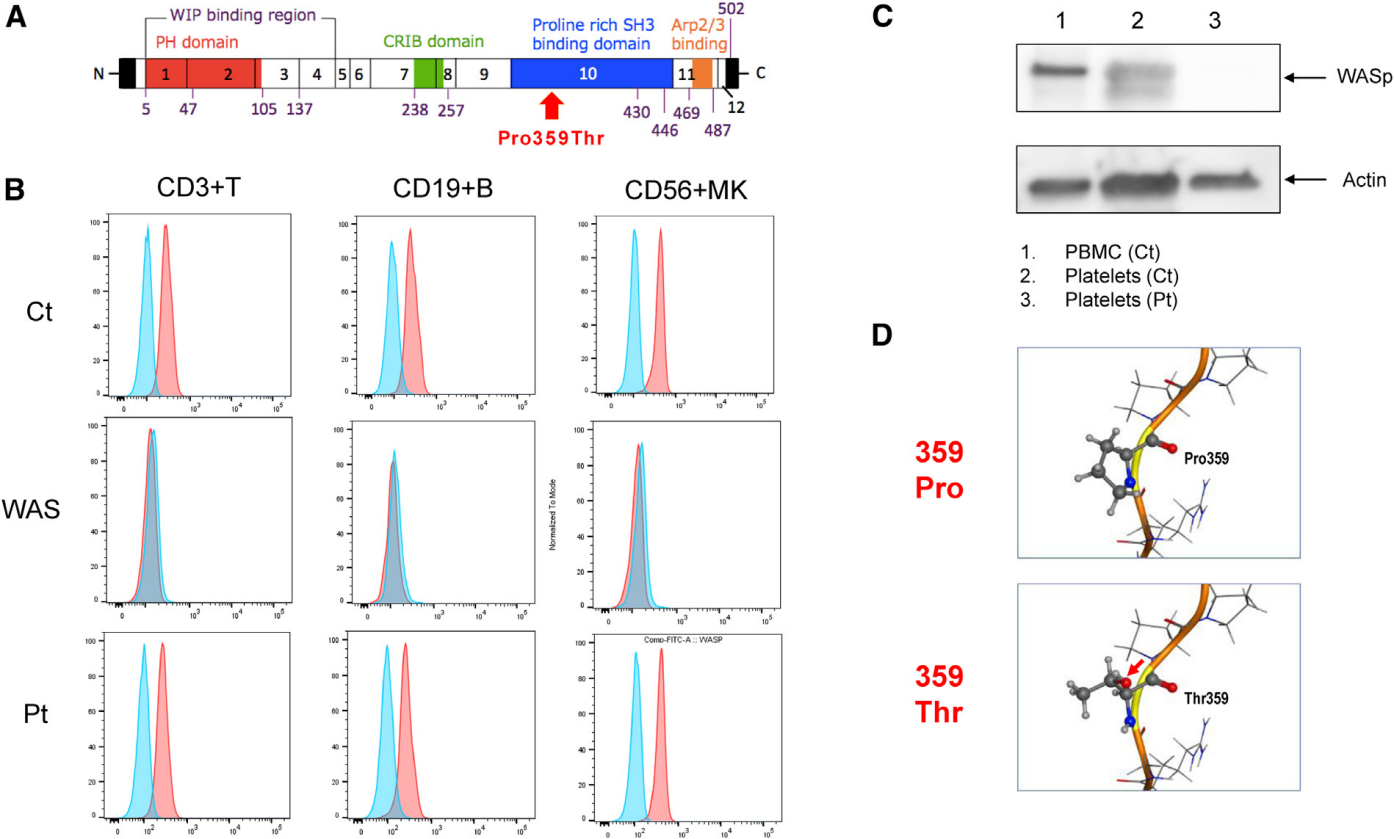
**A,** Position of p.(Pro359Thr) variant in proline-rich PxxP motif of WASp. Pro359 locates in proline cluster region and is conserved between multi-species. The proline-rich domain contains sites for binding of Src homology 3 domain-containing proteins, and the binding of Src kinase stabilizes active WASp via the phosphorylation of tyrosine 291 and follows actin polymerization. **B,** Flow cytometric analysis of WASp in the lymphocyte subsets in a healthy control, a classical WAS case, and this patient. T cells, B cells, and natural killer cells from the patient showed a similar level of WASp expression as those from a healthy control. **C,** Western blot analysis of WASp expression in platelets. WASp was not detected in platelets from the patient. **D,** Protein 3-dimensional structure estimates of Pro359Thr. The substitution of the 359th proline to threonine is suggested to change molecular conformation and polarity of the side chain, leading to a destabilization of the surrounding structure and, consequently, a loss of binding activity. A red arrow indicates new polarity. Ct, healthy control; Pt, patient; PBMC, peripheral blood mononuclear cells; Pro, proline; Thr, threonine.

The results of flow cytometry of WASp expression revealed that all T cells, B cells, and natural killer cells had the same level of WASp expression as healthy controls (Figure, B). In lymphocytes, it has been reported that WASp analysis by flow cytometry is useful compared with WASp analysis by Western blot.^8^ Western blot analysis revealed no expression of WASp in platelets (Figure, C). These clinical and laboratory findings confirmed the diagnosis of XLT.

In the present case, WASp expression in lymphocytes was normal, unlike most cases of XLT. Therefore, a structural analysis of WASp based on the gene variant in this case was performed (Figure, D). Of the amino acids encoded by the *WAS*, proline-359 was substituted by threonine, which changed the charged state and possibly inhibited binding to the SH3 domain, resulting in the obstruction of actin polymerization (Figure, D). These findings could account for the decreased WASp activity in the patient.

This study was approved by the Ethics Committees of the National Center for Child Health and Development in May 2018 (#1818). Informed consent for publishing the findings in this report was obtained from his parents.

## Discussion

In this article, we present the case of a 3-year-old patient with a diagnosis of XLT in whom WASp expression was normal in lymphocytes but absent in platelets. The missense variant of c.1075 C > A, p.(Pro359Thr) found in this case had not been reported previously.

The *WAS* gene encodes WASp and is composed of 12 exons containing 1823 base pairs (Figure, A).^1^ Approximately 300 types of gene variants in the *WAS* gene spanning all 12 exons have been identified, but missense variants are predominantly located in exons 1 to 4 (Figure, A).^9^ Deletions and insertions are distributed throughout *WAS* gene, and splice site variants are predominantly found in introns 6, 8, 9, and 10.^9^ In the present case, a missense variant was found in exon 10, the first instance of a missense variant occurring at this exon.

Like typical cases of XLT, persistent thrombocytopenia developed without eczema or a compromised immune system in the present case. The severity of WAS and XLT-related symptoms is scored on a scale of 1-5 using a WAS disease severity scoring system.^1^ Most patients with a missense variant scored 1 or 2 (XLT phenotype).^1^ In contrast, patients with a nonsense variant or deletions/insertions scored 3 to 5 (the classical WAS phenotype).^1^

Although patients with a missense variant often have lymphocytes with decreased WASp expression, previous studies reported cases with a normal WASp expression in lymphocytes.^1^^,^^10^ In our literature review, there were 5 cases with *WAS* gene variants that showed normal WASp expression in lymphocytes (Table). Two of the 5 were brothers with the same genetic variant, and the others were from different families. In all cases, the variant type was missense, the only symptom was thrombocytopenia, with a severity score of 1 and a clinical phenotype of XLT. Based on our review, XLT cases with normal WASp expression in lymphocytes were clinically mild, as in our case. However, a study investigating the natural course of XLT reported the risk of severe bleeding episodes generally within the first 30 years of life, although the risk of developing autoimmune diseases, malignant tumors, or life-threatening infectious episodes was reported throughout the patient's lifetime. Therefore, there is a possibility that symptoms, including long-term complications, may not be mild.^4^

**Table:** Cases with *WAS* gene variants in which WASp expression were normal in lymphocytes

Patient	Clinical phenotype	Mutation type	Exon	cDNA variant (predicted protein change)	Severity score	WASp in lymphocytes (method of detection)
1,2∗	XLT	Missense	1	52G > A,(Met6Ile)	1, 1	Normal (Western blot)
3∗	XLT	Missense	2	201C > T,(Ala56Val)	1	Normal (Western blot)
4∗	XLT	Missense	2	290 >T,(Arg86Cys)	1	Normal (Western blot)
5†	XLT	Missense	11	1476T > A,(Ile481Asn)	1	Normal (Western blot)

WASp, Wiskott-Aldrich syndrome protein; XLT, X-linked thrombocytopenia.

Patients 1, 2 were brothers.

∗ Reported by Imai et al.^1^

† Reported by Notarangelo et al.^10^

The mechanism by which WASp expression in lymphocytes is normal and WASp expression in platelets disappears when these missense variants are present is unknown. Pro359 is present within the SH3-binding domain on the C-terminal side of WASp. Pro359 is conserved in mammals and other species and is also a key amino acid constituting the XPXXP motif,^11^ which is important for binding to the SH3 domain. Pro359Thr is presumed to reduce or lose its SH3 domain binding activity.^12^ The 3-dimensional structures of the wild-type and mutant WASps were estimated using AlphaFold2 data (https://alphafold.ebi.ac.uk/entry/P42768) and the Molecular Operating Environment software (MOLSIS Inc., Tokyo, Japan), as previously described.^13^ It was estimated that the substitution of the 359th proline to threonine would result in a change in the molecular conformation and polarity of the side chain, leading to a destabilization of the surrounding structure and, consequently, a loss of binding activity (Figure, D). The reason for normal WASp expression in lymphocytes but its absence in platelets remains unclear. One hypothesis suggested differences in WASp expression between these cell types.^14^ In WAS/XLT, as observed in this case, WASp is always deficient in platelets.^14^ Enhanced protein degradation in platelets is hypothesized to underlie this deficiency.^14^ The presence of distinct proteases in platelets compared with lymphocytes, combined with minimal protein synthesis (protein replacement) levels in platelets, could potentially contribute to proteolytic defects.^14^ To identify this hypothesis and to determine why WASp levels remain normal in lymphocytes, further molecular biological research is required, considering proteases and other factors.

## Conclusions

We report a patient with XLT with normal WASp expression in lymphocytes and a novel gene variant. Analysis of WASp in both lymphocytes and platelets could increase the sensitivity of the diagnosis of XLT in pediatric patients with chronic thrombocytopenia.

## Declaration of Competing Interest

Supported by grants to **A.I.** from the 10.13039/100009619Japan Agency for Medical Research and Development (18ek0109366h0001, 19ek0109366h0002, and 20ek0109366h003) and the 10.13039/100007786National Center for Child Health and Development (2021-B3). The authors have nothing to disclose.

## CRediT authorship contribution statement

**Serena Hamanaka:** Writing – original draft. **Toru Uchiyama:** Writing – review & editing, Supervision, Investigation. **Tadashi Kaname:** Writing – review & editing, Supervision, Investigation. **Motohiro Matsui:** Writing – review & editing, Supervision, Conceptualization. **Hiroshi Yoshihashi:** Writing – review & editing, Supervision. **Atsushi Makimoto:** Writing – review & editing, Supervision, Conceptualization. **Yuki Yuza:** Writing – review & editing, Supervision. **Akira Ishiguro:** Writing – review & editing, Supervision, Conceptualization.
